# Co^II^(Chromomycin)_2_ Complex Induces a Conformational Change of CCG Repeats from i-Motif to Base-Extruded DNA Duplex

**DOI:** 10.3390/ijms19092796

**Published:** 2018-09-17

**Authors:** Yu-Wen Chen, Roshan Satange, Pei-Ching Wu, Cyong-Ru Jhan, Chung-ke Chang, Kuang-Ren Chung, Michael J. Waring, Sheng-Wei Lin, Li-Ching Hsieh, Ming-Hon Hou

**Affiliations:** 1 Institute of Biotechnology, National Chung-Hsing University, Taichung 402, Taiwan; dodochinchin@gmail.com; 2 Institute of Genomics and Bioinformatics, National Chung-Hsing University, Taichung 402, Taiwan; roshan.satange@gmail.com (R.S.); jane871057@gmail.com (P.-C.W.); 3 Ph.D. Program in Medical Biotechnology, National Chung Hsing University, Taichung 402, Taiwan; 4 Department of Life Sciences, National Chung-Hsing University, Taichung 402, Taiwan; f810409@gmail.com; 5 Institute of Biomedical Sciences, Academia Sinica, Taipei 115, Taiwan; chungke@ibms.sinica.edu.tw; 6 Department of Plant Pathology, National Chung-Hsing University, Taichung 402, Taiwan; krchung@nchu.edu.tw; 7 Department of Biochemistry, University of Cambridge, Cambridge CB2 1GA, UK; mjw11@cam.ac.uk; 8 Institute of Biological Chemistry, Academia Sinica, Taipei 115, Taiwan; sanway@gate.sinica.edu.tw; 9 Advanced Plant Biotechnology Center, National Chung-Hsing University, Taichung 402, Taiwan

**Keywords:** i-motif, CCG repeats, trinucleotide repeat DNA, chromomycin A3, neurological disease, X-ray crystallography, nucleotide flip-out, DNA deformation

## Abstract

We have reported the propensity of a DNA sequence containing CCG repeats to form a stable i-motif tetraplex structure in the absence of ligands. Here we show that an i-motif DNA sequence may transition to a base-extruded duplex structure with a GGCC tetranucleotide tract when bound to the (Co^II^)-mediated dimer of chromomycin A3, Co^II^(Chro)_2_. Biophysical experiments reveal that CCG trinucleotide repeats provide favorable binding sites for Co^II^(Chro)_2_. In addition, water hydration and divalent metal ion (Co^II^) interactions also play a crucial role in the stabilization of CCG trinucleotide repeats (TNRs). Our data furnish useful structural information for the design of novel therapeutic strategies to treat neurological diseases caused by repeat expansions.

## 1. Introduction

The formation of expanded repeat sequences has long been known to correlate with the etiology of many human diseases [1,2,3]. Tandem repeats can form unusual DNA structures, resulting in consecutive GpC sites that are flanked by mismatched G:G or C:C base pairs in the X chromosome [2,4,5]. Fragile X syndrome (FXS) is a genetic disorder caused by an expansion of CGG/CCG tandem repeats in the Fragile X Mental Retardation 1 gene (*FMR1*) on the X chromosome [6,7]. The repeats in *FMR1* result in a defective protein that has been associated with symptoms of FXS. Recently, our studies have suggested that the expansion of (CCG)_n_ trinucleotide repeats (TNRs) may be attributed to the slippage of DNA strands along the hairpin structures, forming a four-stranded helical structure that is stabilized by intertwining i-motifs during DNA replication [8].

Small molecules that specifically bind to TNR DNA conformations could have applications as diagnostic tools as well as therapeutic agents against these genetic diseases. For example, naphthyridine derivatives can inhibit DNA polymerases during replication because they can selectively recognize and stabilize the CNG repeat hairpin structures formed by a single-strand DNA expansion [9]. Moreover, several well-known DNA-binding drugs including actinomycin D, doxorubicin and mitomycin C have been demonstrated to prevent the amplification of abnormal CNG trinucleotide repeats [10,11,12]. Chromomycin A3 (Chro), produced by some strains of *Streptomyces griseus*, is an anthraquinone glycoside antibiotic belonging to the aureolic acid family [13]. Chro contains di- and trisaccharide components linked to a β-ketophenol chromophore (anthracene ring) via *O*-glycosidic bonds at position 2 and 6, respectively. Chro can bind to divalent metal ions and form a dimer, (Chro)_2_, that has a unique fluorescent emission under different environmental conditions [14]. Previously, Ni^II^(Chro)_2_ has been shown to bind specifically to CCG TNRs via a “forced” induced-fit mechanism [15]. Upon binding to TNRs, Ni^II^(Chro)_2_ exhibits a unique fluorescence signature which can potentially be used to identify fragile X syndrome in clinical specimens.

However, previous studies utilized different DNA sequences, prompting the question of whether chromomycin compounds are capable of “transforming” an i-motif sequence to a base-extruded sequence. The identity of the metal ion might also be significant; e.g., Ni may or may not be important for binding. In the presence of divalent cobalt ions, Chro can also form a metal-coordinated dimer Co^II^(Chro)_2_, which binds selectively to GpC sequences in the minor groove of DNA. In the current study, experiments were conducted to gain a better understanding of the effects of Co^II^(Chro)_2_ on the i-motif structure of CCG TNRs. The crystal structure of the dT(CCG)_3_A sequence has been solved in the presence and absence of the cobalt-containing Chro dimer. These studies revealed that the CCG repeats can fold into a hairpin structure with tetraplex i-motif formation. Co^II^(Chro)_2_ can alter hairpin formation of CCG repeat DNA and is responsible for the formation of a double-helical conformation of CCG repeat DNA with dual cytosine flipping. The results also revealed that water-mediated interactions and divalent cobalt ions are essential to maintain the conformational integrity and stability of the Chro-DNA complex.

## 2. Results

### 2.1. A Non-Canonical DNA Structure of the dT(CCG)_3_A Sequence Contains an i-Motif Tetraplex Core

**Figure 1:**
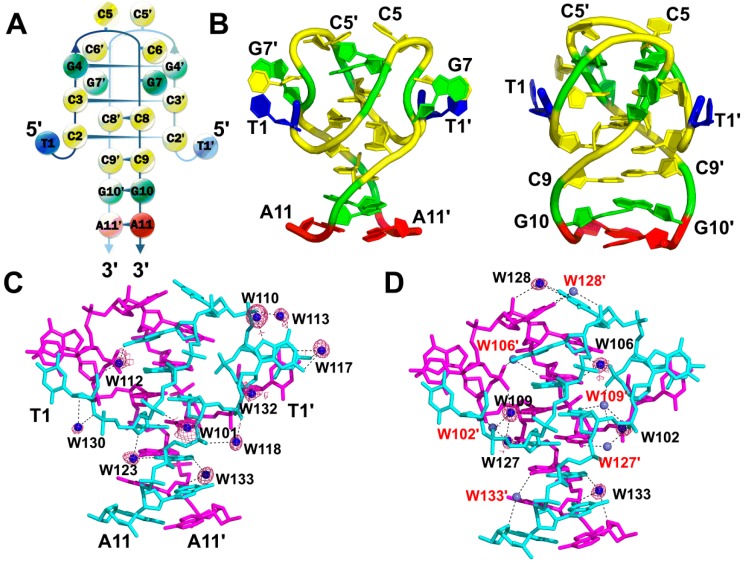
The structural features of dT(CCG)_3_A i-motifs: (**A**) Schematic diagram of the crystal structure of two symmetrical dT(CCG)_3_A strands that fold into a tetraplex i-motif as shown on the left. Guanine bases are colored in green, adenine in red, thymine in blue, and cytosine in yellow. (**B**) Representation of the dT(CCG)_3_A final refined structure viewed from the narrow-groove (middle) and wide-groove (right) directions. (**C**) Inter-strand water cluster stabilizing the single-stranded hairpin structure. (**D**) View of the water cluster formed between two hairpins of dT(CCG)_3_A from the narrow groove. Each DNA strand is colored magenta or cyan. *2Fo-Fc* electron density map of the coordinated waters (blue spheres) in the refined structure is contoured at 1.0 σ, while the waters coordinating from the other asymmetric unit are shown as slate-coloured spheres. The hydrogen bonds are represented by dashed lines within the distance of 3.5 Å.

The dT(CCG)_3_A sequence in the absence of Co^II^(Chro)_2_ was crystallized in a slightly acidic environment (pH 6.0) to yield a high resolution structure of 1.71 Å. The initial phase for the dT(CCG)_3_A was solved using the previously reported coordinates of PDB ID: 4PZQ. All atoms present in the DNA molecule were included in the refined structure and exhibited clear electron density, as shown in the Supplementary Materials Figure S1A. The crystal structure revealed that each asymmetric unit contained a single-strand dT(CCG)_3_A molecule, which could form a CCG loop by folding back within the central CCG unit to generate a hairpin-like structure (Figure 1A,B). Two symmetrical dT(CCG)_3_A hairpins joined together by hydrogen bonds to form a tetraplex structure with an i-motif core, which includes four intercalated C:C^+^ base pairs flanked by two G:G homopurine base pairs. Moreover, several stacking interactions were observed in the two symmetrical dT(CCG)_3_A hairpins, which were important for maintenance of the i-motif conformation. These stacking interactions included a 5′ cytosine residue (C5), which protruded into the centre of the i-motif core to form a stacking interaction with G4 of the other strand. Two flipped-out nucleotides (C6 and G7) in each of the central CCG loops stacked together with the 5′-end T1 base, which was tilted out into the wide groove. The 3′ ends of two CGA oligonucleotides were aligned in parallel, resulting in C:C^+^, G:G^+^ and A:A^+^ base pairs forming a right-handed duplex stem. Along with overwound twist angles at the G4–C3 and C9–G10 steps, CGA oligonucleotides formed two symmetrical hairpins that were tightly twisted in a clockwise direction to produce a right-handed tetrahelix.

### 2.2. Stabilization of the i-Motif Tetraplex by Water Hydration

In total, fifteen bridging water molecules were identified as mediating the interactions between the two hairpin structures of dT(CCG)_3_A (Figure 1C,D). Six water molecules (W102, W106, W109, W127, W128, and W133) mediated the DNA-DNA inter-strand interactions, while ten water molecules (W101, W110, W112, W113, W117, W118, W123, W130, W132 and W133) mediated the DNA-DNA intra-strand interactions. Interestingly, W133 was found to mediate both inter- and intra-strand interactions. W110, W113, and W117 stabilized the central CCG loop structure. Two flipped-out C5 bases in opposite dT(CCG)_3_A strands were linked by W128 located at the top of the structure. W102, W109 and W127 water molecules mediated the interactions between the cytosine residues (C2 and C8) at the i-motif core. W106 linked the pyrimidine base C3 and the purine base G4 by bridging the N2 of G4 and the O2 of C3 in the opposite chain. Moreover, W133 played a key role in stabilizing the structure by mediating the inter-strand G10-A11 interaction as well as inter-strand C9-G10 interaction of the dT(CCG)_3_A hairpin structure. Analysis of the high-resolution crystal structure of the i-motif tetraplex revealed that water was positioned so as to hold and stabilize the dimeric hairpins via hydrogen bonds. A list of intra- and inter-strand water-mediated interactions with their respective distances between the atoms in the two symmetrical dT(CCG)_3_A hairpins is provided in Table S2. Interestingly, the structure reported here did not involve metal ions as reported in the previous structure [8], instead relying exclusively on water-mediated interactions to stabilize the i-motif.

### 2.3. Co^II^(Chro)_2_ Complex Induces Conformational Changes in the d[T(CCG)_3_A]_2_ DNA Duplex

**Figure 2:**
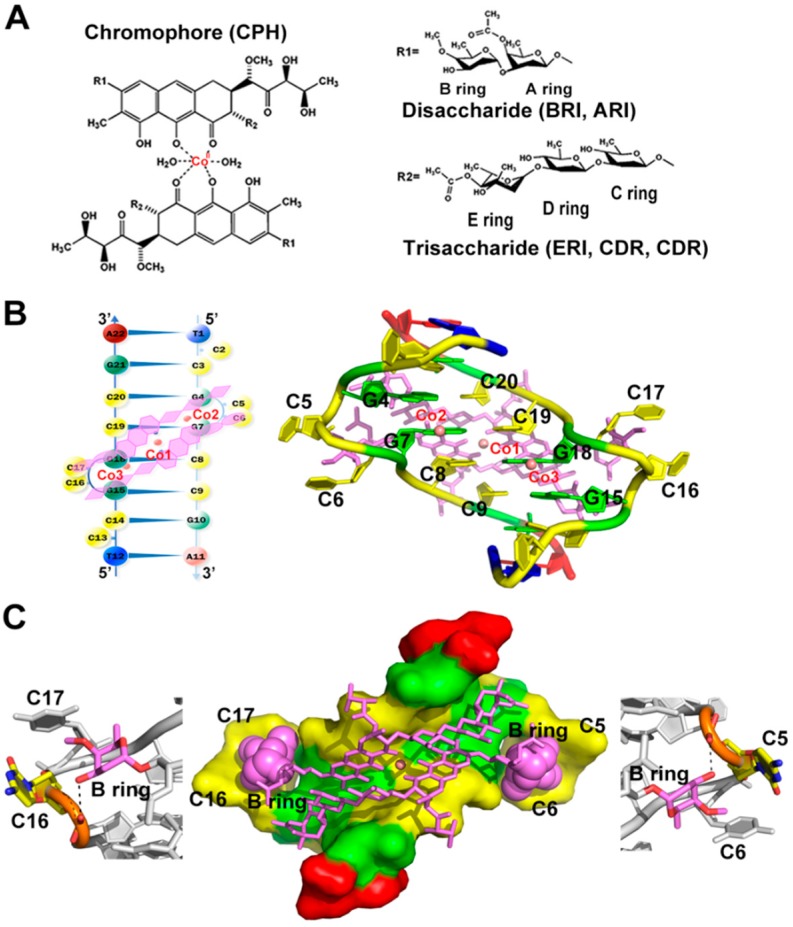
Crystal structure of the Co^II^(Chro)_2_-d[T(CCG)_3_A]_2_ complex. (**A**) Chemical structure of the Co^II^(Chro)_2_ dimer. (**B**) Schematic diagram of the Co^II^(Chro)_2_-d[T(CCG)_3_A]_2_ complex shown on the left. The cobalt(II) ions and the Co^II^(Chro)_2_ complex are drawn in salmon and pink. The refined structure of the Co^II^(Chro)_2_-d[T(CCG)_3_A]_2_ complex viewed from the major groove is shown on the right. The Co^II^(Chro)_2_ complex binds to the central G4-C20, G7-C19, C8-G18 and C9-G15 base pairs of the d[T(CCG)_3_A]_2_ DNA structure accompanied by extrusion of four cytosine bases. (**C**) The extruded cytosine residues stabilized by the disaccharide B ring of (Chro)_2_ via hydrogen bonds are shown at sides with hydrogen bonds indicated by dashed lines. In the middle of the complex, stabilized by van der Waals forces, is represented with the disaccharide B ring and d[T(CCG)_3_A]_2_ duplex viewed from the minor-groove direction (sphere and solvent-accessible surface respectively).

Chro bound to cobalt divalent cations to form a [Co^II^(Chro)_2_] dimer (Figure 2A). To understand the structure of the dT(CCG)_3_A sequence in the presence of dimer, Co^II^(Chro)_2_ bound to the DNA sequence was crystallized in a similar manner to that described for the formation of the dT(CCG)_3_A i-motif crystals. The electron density map with a resolution of 1.87 Å revealed that all atoms in the refinement structure had a clear electron density, except for two cytosines (C2 and C13), which were extruded from the structure due to poor mapping and had to be modelled with an energy minimization module using Accelrys Discovery Studio Client (v2.5.0.9164) (Figure S1C) [16]. The extruded cytosines thus form an e-motif structure which might stabilize the packing of the complex within the crystal lattice. The presence of such structures has been reported previously [17,18,19]. Analysis of the crystal structures revealed that Co^II^(Chro)_2_ altered the formation of the i-motif tetraplex, which was composed of two hairpin-like structures. Co^II^(Chro)_2_ bound to the pseudo-palindromic duplex DNA sequence in the minor groove, resulting in the formation of a Co^II^(Chro)_2_-d[T(CCG)_3_A]_2_ complex (Figure 2B). The binding resulted in deformation of the DNA resembling the Ni^II^(Chro)_2_ dimer compounds. A central d(GGCC) motif was formed due to the extrusion of four cytosines (C5, C6, C16, and C17) upon binding to a Chro dimer. Unlike the i-motif adopted by dT(CCG)_3_A dimeric hairpins, the guanine base of the second CCG unit of each DNA strand was not flipped out, resulting in the re-formation of a GGCC tetranucleotide tract that provided the flexibility in the DNA to better accommodate Co^II^(Chro)_2_. The four projected cytosines (C5, C6, C16, and C17) interacted with the disaccharide B ring of (Chro)_2_ so as to enhance the stability of the extruded residues via hydrogen bonds and van der Waals forces (Figure 2C). We suppose that the observed conformation for the DNA within the complex is effectively induced by Co^II^(Chro)_2_ binding. Although we cannot exclude the possibility that both the i-motif and duplex structure co-exist in solution, and Co^II^(Chro)_2_ may “choose” the duplex structure through conformational selection, we consider the possibility to be low for the following reasons: (1) the i-motif has been shown to be more stable than its duplex counterpart [20] and should be the favoured conformation, and (2) circular dichroism studies have also shown that the CCG repeats adopt spectra indicative of the i-motif conformation in solution [21,22].

### 2.4. Stabilization of the Co^II^(Chro)_2_-d[T(CCG)_3_A]_2_ Complex by Interacting with Cobalt(II) Ions and Water Hydration

**Figure 3:**
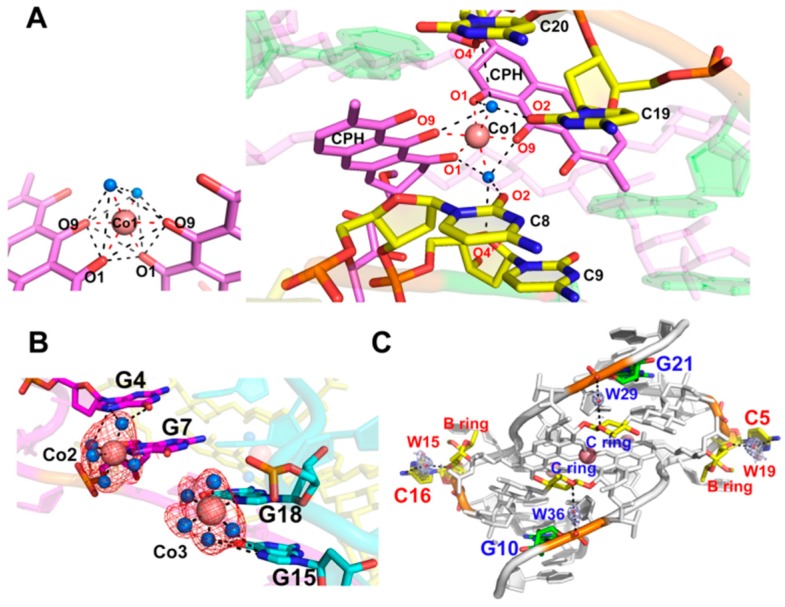
Coordination of cobalt ions and water mediate interactions in the Co^II^(Chro)_2_-d[T(CCG)3A]_2_ complex structure. (**A**) Close-up view from the major groove of the Co^II^(Chro)_2_-d[T(CCG)_3_A]_2_ complex representing the specific interactions between Co^II^(Chro)_2_ and central GGCC steps. The octahedral coordination of Co1 is also shown on the left. The cobalt ion and two water molecules that mediate Chro and DNA interactions are represented as salmon and blue spheres, respectively. Coordination and hydrogen bonds are shown by dashed lines. (**B**) Close-up view of the Co^II^(Chro)_2_-d[T(CCG)_3_A]_2_ structure showing the octahedral coordination of Co2 and Co3 ions interacting with the unpaired N7 [G7] and N7 [G18] bases, respectively, and the coordinated water molecules also involved in mediating the cobalt(II) ions and G4 (G15) base interactions. (**C**) The bridging water molecules mediate the interaction between the Co^II^(Chro) and DNA viewed from the minor groove. The *2Fo-Fc* electron density map is contoured at a 1.0 σ level.

There were three cobalt ions (Co1, Co2, and Co3, Figure 2B) present in the Co^II^(Chro)_2_-d[T(CCG)_3_A]_2_ complex. The Co1 ion formed an octahedral coordination with two oxygen atoms, O1 and O9, of each chromophore moiety and two oxygen atoms of water in the centre of the (Chro)_2_ complex (Figure 3A). The two water oxygen atoms were also involved in the formation of a hydrogen bond network between the cytosine base of the GGCC tetranucleotide tract and the chromophore of the (Chro)_2_ complex. In the major groove, the Co2 and Co3 ions interacted with N7 of guanine G7 and G18, also forming an octahedral coordination with guanine and five water molecules (Figure 3B). Water molecules were further involved in the interactions between the G7 and G4 bases and the G18 and G15 bases. Water bound to the GpG steps of the tetranucleotide tract, which was formed by extruding two cytosine bases of each DNA strand (Figure 3C). Previous studies have shown that water molecules commonly play a profound role in groove binding [23,24]. In the Co^II^(Chro)_2_-d[T(CCG)_3_A]_2_ complex structure, a total of four water molecules were identified as being directly involved in the interaction between Co^II^(Chro)_2_ and the CCG TNRs (Figure 3C). These four water molecules (W15, W19, W29, and W36) stabilized the extruded cytosine residues and the last guanine in the complex via specific water-mediated interactions with (Chro)_2_. W15 and W19 bridged the interactions between the N4 amine on the extruded cytosines and the O3 oxygen atoms of the disaccharide B ring. W29 and W36 formed water-mediated hydrogen bonds with the phosphate oxygen atom of the last guanine (G10 and G21) and the O1 oxygen atoms of the trisaccharide C ring. These interactions mediated by water molecules could also be found in the Ni^II^(Chro)_2_-d[TT(CCG)_3_AA]_2_ complex structure, indicating that they were indispensable for recognition of the (Chro)_2_ ligand by the CCG DNA repeat as well as the stability of the complex.

### 2.5. The Cobalt–Chro Complex Specifically Recognizes the Hairpin Structure of CCG TNRs

**Figure 4:**
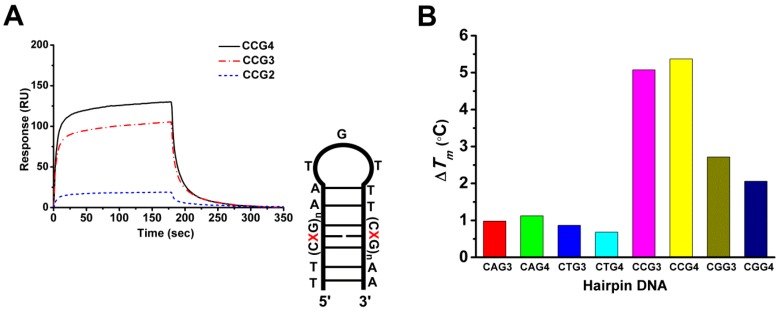
Binding affinity and stabilizing effects of the Co^II^(Chro)_2_ complex on various d(CXG)_n_ trinucleotide repeats, where X could be any base and n is the repeat number, as shown in the schematic diagram. (**A**) Surface plasmon resonance (SPR) sensorgrams representing the binding of the Co^II^(Chro)_2_ complex to the immobilized 5′ biotin-labelled hairpin DNAs (CCG2, CCG3, and CCG4). The reactions were carried out in 50 mM sodium cacodylate buffer (pH 7.3) containing 50 mM NaCl. The resonance unit (RU) is defined as 1 RU = 1 pg/mm^2^. (**B**) Effects of Co^II^(Chro)_2_ on the *T_m_* values of various hairpin DNA fragments measured in 50 mM sodium cacodylate buffer (pH 7.3) containing 50 mM NaCl, with DNA and Co^II^(Chro)_2_ at a 1:4 molar ratio. ∆*T_m_* values were obtained by subtracting the *T_m_* value in the presence of the ligand.

Surface plasmon resonance (SPR) was employed to analyse the binding affinity of Co^II^(Chro)_2_ to d(CCG)_n_. The results revealed that the interactions of Co^II^(Chro)_2_ with d(CCG)_3_ or d(CCG)_4_ resulted in a high resonance unit (RU) (Figure 4A), which is indicative of strong binding. However, Co^II^(Chro)_2_ failed to bind to d(CCG)_2_, resulting in a low RU (Figure S2). The binding rate constant (*k_a_*) and the dissociation rate constant (*k_d_*) were calculated according to the kinetic 1:1 Langmuir binding model, and the binding constant (*K_a_*) was calculated from the values of *k_a_* and *k_d_* respectively (Table 1). We found that the binding affinity of Co^II^(Chro)_2_ for DNA could be enhanced by increasing the number of CCG repeat units. To determine the selectivity and stabilization effects of Co^II^(Chro)_2_ on various TNR DNA sequences, the melting temperature differences (∆*T_m_*) of a duplex DNA were measured. Various d(CXG)_n_ repeats, where X could be any base and n was the repeat number, were synthesized and used for binding assays with Co^II^(Chro)_2_ at a ratio of 1:4. The results indicated that the binding of Co^II^(Chro)_2_ to d(CXG)_n_ increases the overall stability of d(CXG)_n_ compared to controls. The type of trinucleotide repeat also considerably impacted ∆*T_m_* (Figure 4B). The binding of Co^II^(Chro)_2_ to d(CAG)_3or4_ or d(CTG)_3or4_ resulted in a low ∆*T_m_*, indicative of a poor stabilization effect. By contrast, binding Co^II^(Chro)_2_ to d(CCG)_3or4_ or d(CGG)_3or4_ resulted in high ∆*T_m_* values. It appears that Co^II^(Chro)_2_ binds preferentially to d(CCG)_3or4_ duplexes compared to other d(CXG)_n_ repeats, clearly indicating the selective ligand binding.

## 3. Discussion

**Figure 5:**
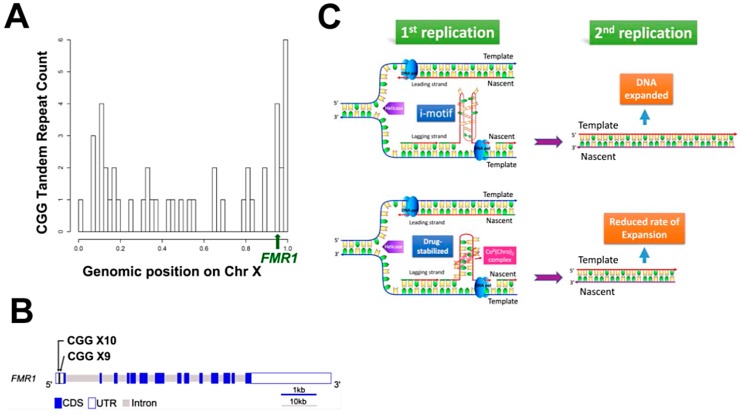
CGG/CCG tandem repeats on the *FMR1* gene and DNA expansion. (**A**) The distribution of the CGG/CCG tandem repeats with more than five on chromosome X and the location of the *FMR1* gene (the length of chromosome X scaled to 1). There are several CGG/CCG tandem repeats around the locations of the *FMR1* gene in the normal human genome. (**B**) The gene structure for human *FMR1*. There are 2 CGG/CCG tandem repeats with 9 and 10 copies, respectively, found on the 5′ UTR of *FMR1* in the normal human genome. (**C**) A proposed model for the biological consequences that occur following binding of the Co^II^(Chro)_2_ complex to the slipped CCG repeats at the nascent lagging strand during DNA replication. In the first cycle of DNA replication, if slipped CCG repeats fold into an unstable tetraplex i-motif structure on the nascent strand, it would lead to a subsequent expansion when using the nascent lagging strand as a template in the next cycle of replication. In contrast, when the Co^II^(Chro)_2_ complex recognizes the slipped CCG repeats, the resulting DNA conformational change may help to stabilize the slipped DNA and lead to the newly generated DNA duplex, with no length changes in the next cycle of DNA replication.

Fragile X syndrome (FXS) is a genetic disorder caused by the expansion of CGG/CCG tandem repeats in the Fragile X Mental Retardation 1 gene (*FMR1*) on the X chromosome [6,25]. The CGG/CCG tandem repeats are scattered along the X chromosome, with a high percentage around *FMR1* (Figure 5A). Examination of the coding and noncoding sequences of *FMR1* revealed the presence of two CGG/CCG tandem repeats with 9 or 10 copies in the 5′ untranslated region (UTR) (Figure 5B). No tandem repeats were found in the coding region. The expansion of repeats in *FMR1* results in a defective protein that is known to be associated with the symptoms of FXS [26]. Previous studies have shown that under physiological conditions, both the G-rich and C-rich single-stranded d(CGG)·d(CCG) repeats are able to form secondary structures and cause unusual expansions [8,27,28]. The propensity to form the secondary structure is more pronounced for d(CCG)_n_ than it is for d(CGG)_n_ repeats [29]. This observation suggests that the d(CCG)_n_ strand is more likely to form a hairpin or slippage structure and exhibit an asymmetric strand expansion during DNA replication. In addition to hairpin structures, CCG repeats have been reported to adopt a tetraplex structure based on two parallel-oriented hairpins that are held together by hemiprotonated intermolecular C:C+ pairs [30].

Recently, researchers have focused on designing novel compounds that can bind to expanded CNG repeat DNA in a sequence-specific manner. The propensity of small molecules to bind to the substrate with expanded CNG repeats can inhibit abnormal DNA replication as well as transcription [31,32,33]. In the present study, we have found that two d(T(CCG)_3_A)_2_ sequences can form hairpin structures that stack together in parallel to form a tetraplex, with a core i-motif surrounded by two G:G homo-base pairs. A similar structure has been reported previously in which it was shown that divalent cobalt ions are crucial for maintaining the i-motif structure [8]. However, the i-motifs reported in this work are exclusively stabilized by water molecules. Many of the water molecules occupy similar positions to the cobalt ions observed in the previous work. Furthermore, we observed here that several interactions, including stacking forces, stabilize the extruded residues in the i-motif tetraplex. On the other hand, the structure of two d(T(CCG)_3_A)_2_ sequences in the presence of the Co^II^(Chro)_2_ complex clearly shows a double-stranded helical conformation, which implies a crucial role of Co^II^(Chro)_2_ in binding to CCG. Evidently, the binding of Co^II^(Chro)_2_ to the d(T(CCG)_3_A)_2_ causes the extrusion of four cytosines in the repeat motif. It is conspicuous that the chromomycin A3 restores the double-stranded helical conformation by preventing the repeat DNA motif from forming i-motif structures (Figure S3).

Previously, the Ni^II^(Chro)_2_ complex has been shown to bind specifically to CCG TNRs via a “forced” induced-fit mechanism [15]. The Co^II^(Chro)_2_ complex structure with DNA shows significant similarity in overall structure with that of the Ni^II^(Chro)_2_ complex. However, the stability of these structures in terms of melting temperature is quite different, which clearly implies the occurrence of local differences. Analysis of the central GpGpCpC segment surrounding the Chro dimer binding site revealed considerable differences between the helical parameters of the Ni^II^ and Co^II^ complexes. Although the unwinding parameters are similar for both structures, prominent variations in the DNA roll and rise parameters were observed for the central GpC step (Figure S4) [34]. Based on detailed analysis of the structures, we propose a model to explain how tandem repeats could lead to sequence expansion during DNA replication (Figure 5C). The binding of the Co^II^(Chro)_2_ complex to the slipped CCG repeats between the nascent and lagging strands during DNA replication could result in two different situations. If the CCG repeats fold into a tetraplex i-motif structure in the absence of the Co^II^(Chro)_2_ complex during the first DNA replication cycle, DNA polymerase would use the nascent lagging strand as a template for DNA synthesis, leading to sequence expansion during the second replication cycle. On the other hand, recognition and binding of Co^II^(Chro)_2_ complex to the slipped CCG repeats would change the DNA conformation, which might slow down the rate of DNA expansion in the newly generated DNA duplex. After DNA unwinding during replication or transcription, the two strands become separated. Each single strand possesses CGG and CCG repeats, and the C-rich ends may again form i-motif structures consisting of double hairpins. This provides Co^II^(Chro)_2_ with binding sites on both single-strand DNA, thus further reducing the rate of DNA slippage and consequent DNA expansion. The CCG-Co^II^(Chro)_2_ complex, therefore, could prevent the formation of i-motifs and force the DNA to return to a double helical form.

Metal ions often play an important role in either stabilizing DNA structures or stabilizing crystals [35,36]. Many studies have provided detailed information about the interactions of divalent metal ions stabilizing DNA duplexes [37,38,39,40]. Previous work has also highlighted the role of Co(II) metal ions in stabilizing drug-DNA complexes [41]. The divalent cations form a tetrahedral or octahedral coordination complexes with the drug chromophore. It has also been shown that the N7 atom of the purine or N3 of pyrimidine residues as well as exocyclic oxygen atoms and the phosphate O atoms are the preferential sites of metal binding to stabilize the DNA structures in the complex. Furthermore, other divalent metal ions, such as Cu(II) and Hg(II), are also known to alter DNA conformation [42,43]. In the current study, we found that two Co(II) ions can interact with the DNA backbone to stabilize the overall structure. The two metal ions formed coordination bonds with the G7-N7/G18-N7 and five water molecules in a six-coordinate octahedral geometry. The coordinated water molecules were also involved in mediating the interactions between cobalt(II) ions and the G4 (G15) base. Hydrogen bonding between the water molecules and the oxygen atoms of the phosphate backbone can also stabilize the DNA. Based on these interactions, it can be concluded that the inclusion of Co(II) is crucial for maintaining the Co^II^(Chro)_2_-d(T(CCG)_3_A)_2_ complex structure. Since Co^II^(Chro)_2_ has been shown to be less toxic to cells compared to the more potent Ni^II^(Chro)_2_, it may represent a viable alternative for chromomycin-based drug appropriation or development. In addition, the DNA-Co^II^(Chro)_2_ complex has been shown to display extreme resistance to polyamine-mediated extraction of the divalent cation, making it an attractive ligand for exploring its therapeutic potential against CCG-repeat diseases. The potential of other types of metal complexes, such as ruthenium-based ligands, opens up the possibility of studying the interaction of the complexes with unusual DNA structures including i-motifs, triplexes and quadruplexes in future [44].

With this work, we complete our overview of chromomycin-based ligands containing a transition metal ion and their binding effects on repetitive DNA [15,33,45,46]. Our results provide additional clues to piece together the complete flow of how CCG DNA repeat amplification may arise, and provide a structural basis to speed the development and screening of specific drugs to treat diseases caused by the abnormal expansion of repeat DNA motifs.

## 4. Materials and Methods

### 4.1. Chemicals and Oligonucleotides

All chemicals used were of reagent grade, obtained from Sigma Chemical Co. (St. Louis, MO, USA). Deionised water from a Milli-Q system was used for all experimental procedures. Absorbance measurements to determine oligonucleotide concentrations were performed in quartz cuvettes using a Hitachi U-2000 spectrophotometer. The oligonucleotide concentrations were determined by Beer’s law (A = ε.b.c; A: optical density at 260 nm; ε: extinction coefficient; b: cell path length, 1 cm; and c: DNA concentration in M). Synthetic oligodeoxynucleotides were purified by gel electrophoresis. The oligomer extinction coefficients were calculated on the basis of tabulated values for monomer and dimer extinction coefficients with reasonable assumptions as specified in ref. [47].

### 4.2. Melting Temperature Measurements

*T_m_* values for the i-motif tetraplex sequence and the hairpin DNAs complexed with Co^II^(Chro)_2_ were determined as previously described using a JASCO UV-VIS spectrophotometer to monitor the sample absorbance (O.D.) at 260 nm and 295 nm [14,48]. The hairpin DNA, d(TT(CXG)_3-4_AATGTTT(CXG)_3-4_AA, (X = A, T, C, or G), purified from polyacrylamide gel, was the substrate for the *T_m_* experiments (the hairpin loop is underlined). The experiments were performed by increasing the temperature from 5 to 95 °C at a rate of 0.5 °C/min and recording the temperature every 30 s. *T_m_* values (temperature corresponding to the dissociation of half of the DNA structures) were determined from polynomial fitting of the observed curves. The first derivative of the absorbance with respect to the temperature (dA/dT) of the melting curve was computed and used to determine the *T_m_* value.

### 4.3. SPR Analysis

The affinity, association and dissociation between the drug and the DNA duplexes were measured using a BIAcore 3000 A surface plasmon resonance (SPR) instrument (Pharmacia, Uppsala, Sweden) equipped with a sensor chip SA5 from Pharmacia that monitored changes in the refractive index at the surface of the sensor chip. These changes were generally assumed to be proportional to the mass of the molecules bound to the chip and are recorded in resonance units (RU) [49]. The 5′-biotin-labelled hairpin DNA, biotin-d(TT(CCG)_2-4_AATGTTT(CCG)_2-4_AA), purified from polyacrylamide gel electrophoresis, was used in the SPR experiments (the hairpin loop is underlined). To control the amount of DNA bound to the chip surface, the biotinylated oligomer was manually immobilized onto the surface of a streptavidin chip. Solutions of the metal-derived Chro complexes buffered with 50 mM sodium cacodylate at pH 7.3 in 50 mM NaCl were used. Different concentrations of the complexes were passed over the surface of the chip for 180 s at a flow rate of 10 μL min^−1^ to reach equilibrium; one of the flow cells remained blank as a control. Blank buffer solution was then passed over the chip to initiate the dissociation reaction, and this procedure was continued for 300 s to complete the reaction. The surface was then recovered by washing it with 10 μL of a 10 mM HCl solution. The sensorgrams for the interactions between the hairpin DNA duplex and the drug were analysed using version 3 of the BIAcore evaluation software.

### 4.4. Crystallization of d(T(CCG)_3_A) and Co^II^(Chro)_2_-d[T(CCG)_3_A]_2_ Complex

Crystals yielding both the structures of the d(T(CCG)_3_A) i-motif and Co^II^(Chro)_2_-d[T(CCG)_3_A]_2_ complex were obtained in a similar fashion. Both were crystallized using the sitting drop vapour diffusion method. The crystals of d(T(CCG)_3_A) were obtained from a solution of 1.0 mM single-stranded DNA, 50 mM sodium cacodylate (pH 6.0), 1 mM magnesium chloride, 3% 2-methylpentane-2,4-diol, and 0.5 mM cobalt(II) chloride. The solution for crystallization was equilibrated against 500 μL of 30% MPD at 4 °C. Cylinder-shaped crystals of d(T(CCG)_3_A) appeared after 4 weeks. Crystals of the Co^II^(Chro)_2_-d[T(CCG)_3_A]_2_ complex were grown by co-crystallizing 0.75 mM single-stranded DNA, 1.5 mM Chro, and 3 mM CoCl_2_, in 50 mM sodium cacodylate buffer (pH 6.0), 1 mM MgCl_2_, 1 mM spermine and 1% MPD, equilibrated against 500 μL of 30% MPD. Because Chro is yellow, the yellowish colour and the rod-shaped morphology of the crystals implied formation of the d[(TT(CCG)_3_AA)]_2_-Co^II^(Chro)_2_ complex; these were harvested after 4 weeks.

### 4.5. Data Collection, Processing, and Refinement of d(T(CCG)_3_A) and Co^II^(Chro)_2_-d[T(CCG)_3_A]_2_ Complex Structures

The diffraction data for the d(T(CCG)_3_A) crystal in space group *P*4_3_2_1_2 with unit-cell parameters a = b = 38.23, c = 54.23 Å, were collected at 110 K on an ADSC Q315r detector at beamline 13B1 of the National Synchrotron Radiation Research Center (Taiwan). The software package HKL2000 was used to index, integrate, and scale the X-ray diffraction data [50]. The reported resolution of 1.71 Å, at which the structure was refined, is based on the correlation coefficient (CC*) between the data and the model (PDB ID: 4PZQ) using the *PHENIX* suite (v1.8.4-1496) [51]. The nucleotides in d(T(CCG)_3_A) are numbered from T1 to A11 in each strand. The structure was refined using the Refmac5 program in the CCP4 suite [52]. The DNA force field parameters reported by Parkinson et al. were used [53]. The diffraction data in space group *P*3_2_12 with unit-cell parameters a = b = 46.4, c = 73.8 Å, for the d[(T(CCG)_3_A)]_2_-Co^II^(Chro)_2_ complex crystal were collected using the same equipment at 100 K. Fluorescence scanning revealed a strong peak at the Co^II^ wavelength, consistent with the presence of Co^II^ ions in the structure. Multiple-wavelength anomalous diffraction (MAD) data were collected from three wavelengths using cobalt as the anomalous scattering atom. The diffraction spots were indexed, integrated and scaled using the HKL-2000 software package, followed by Co^II^ substructure localization using SHELX C/D/E. The resulting well-defined MAD electron density maps were used to build initial models using the program Coot. These structures were refined using the *PHENIX* program (v1.8.4-1496) using the high remote wavelength data for subsequent refinements. Most of the atoms in the structure were well-resolved and readily assigned in the density map, revealing a clear conformation of the DNA duplex in complex with Co^II^(Chro)_2_, except the uninterpretable region of the bases at the 5′ end of the DNA. B-factor analysis also suggested that the bases at the 5′ end of DNA were thermally less well ordered. Moreover, a well-defined MAD electron density map at a resolution of 1.87 Å was used to build the initial models for d(T(CCG)_3_A) DNA alone. The force field of Chro was generated using the atomic coordinates of a 0.89 Å resolution crystal structure of Co^II^(Chro)_2_. The DNA nucleotide geometry parameters reported by Parkinson et al. were used. The full data collection and refinement statistics are given in Table S1. Coordinates and experimental data can be downloaded from www.wwpdb.org using the PDB IDs in Table S1.

### 4.6. Bioinformatics Analysis

The human genome sequence and the accompanying information pertaining to the gene structure of human *FMR1* were obtained from Ensembl version 91 (with genome assembly GRCh38.p10). We developed in-house software to search the sequence pattern including not less than six CGG tandem repeats against the chromosome X sequence. Statistical analysis was conducted using R Statistical Software (version 3.5.1) (R Foundation for Statistical Computing, Vienna, Austria) [54]. The *FMR1* gene structure was displayed using GSDS 2.0 [55].

## 5. Conclusions

In this report we have demonstrated the propensity of CCG repeats to undergo base pairing between the hemiprotonated cytosine residues of one C-rich hairpin duplex and the cytosine residues of a second hairpin duplex to form a stable i-motif tetraplex structure. The i-motif tetraplex was found to be stabilized by water molecules. The formation of i-motif tetramers may lead to DNA expansion during replication due to the presence of both matched and mismatched base pairs in the CCG repeat region in hairpin or slipped structures. In addition, we found that specific binding of Co^II^(Chro)_2_ to d(CCG)_n_ sequences induced conformational changes of the CCG repeat DNA from i-motif to DNA duplex with cytosine-cytosine flip out. The specificity of Co^II^(Chro)_2_ towards (CCG)_n_ may be partly due to the intrinsic instability and flexibility of C–C mismatches, sufficient to allow adoption of the geometrically optimal conformation that causes cytosines to extrude out of the helix and form the GGCC tetranucleotide patch. Extending the concept further, we hypothesize that TNR-binding compounds may induce a variety of sequences to form specific cognate structural motif(s) representing a substantial step towards the development of new therapeutic or diagnostic agents to treat these neurological diseases.

## Figures and Tables

**Table 1:** Binding parameters for Co^II^(Chro)_2_ complexes and various (CCG)_n_ trinucleotide repeats.

Drugs	DNA Forms	*k*_a_ (M^−1^s^−1^)	*k*_d_ (s^−1^)	*K*_a_ (M^−1^)
Co^II^(Chro)_2_	CCG2	null ^a^	null ^a^	null ^a^
	CCG3	4.19 × 10^3^	4.54 × 10^−2^	9.12 × 10^4^
	CCG4	8.15 × 10^3^	5.68 × 10^−2^	1.43 × 10^5^

^a^ Undetermined.

## Supplementary Materials

Supplementary materials can be found at http://www.mdpi.com/1422-0067/19/9/2796/s1.

## Abbreviations

TNR	Trinucleotide repeats
*FMR1*	Fragile X Mental Retardation 1 gene
FXS	Fragile X syndrome
SPR	Surface plasmon resonance
RU	Resonance Units
PHENIX	Python-based Hierarchical ENvironment for Integrated Xtallography
