# Supplementary material for: CoII(Chromomycin)2 Complex Induces a Conformational Change of CCG Repeats from i-Motif to Base-Extruded DNA Duplex
Source: Int J Mol Sci. 2018 Sep 17;19(9):2796. doi: 10.3390/ijms19092796 (PMC6164834; doi:10.3390/ijms19092796)
Supplement: Supplementary file 1 [file ijms-19-02796-s001.pdf]

International Journal of
Molecular Sciences

Supplementary Material

Co^{II}(Chromomycin)$_2$ complex induces a conformational change of CCG repeats from i-motif to base-extruded DNA duplex

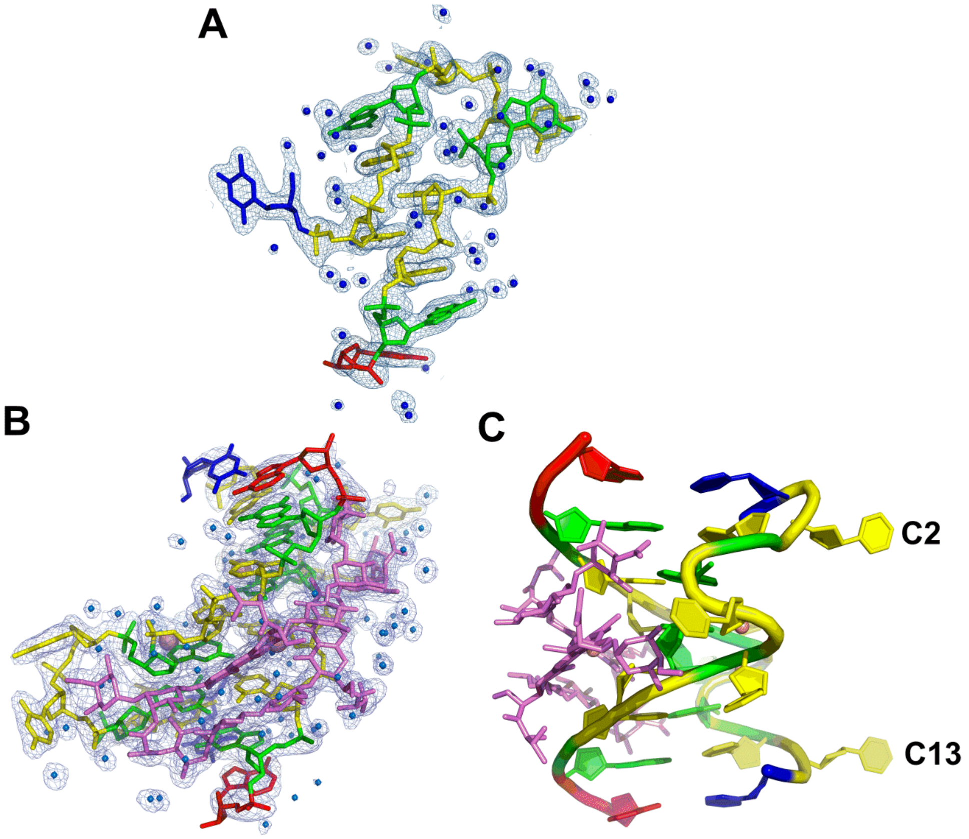

Figure S1. *2Fo – Fc* electron density map of the refined structure of **(A)** dT(CCG)$_3$A and **(B)** Co^{II}(Chro)$_2$-d[T(CCG)$_3$A]$_2$ complex is contoured at the 1.0 σ level. Guanine bases are coloured green, adenine bases are red, thymine bases are blue, cytosine bases are yellow, and Co^{II}(Chro)$_2$ are pink. The cobalt(II) ions and water molecules are represented by salmon and blue spheres, respectively. **(C)** The two extruded cytosines (C2 and C13) without clear electron density maps in the refined structure were modelled by discovery studio software.

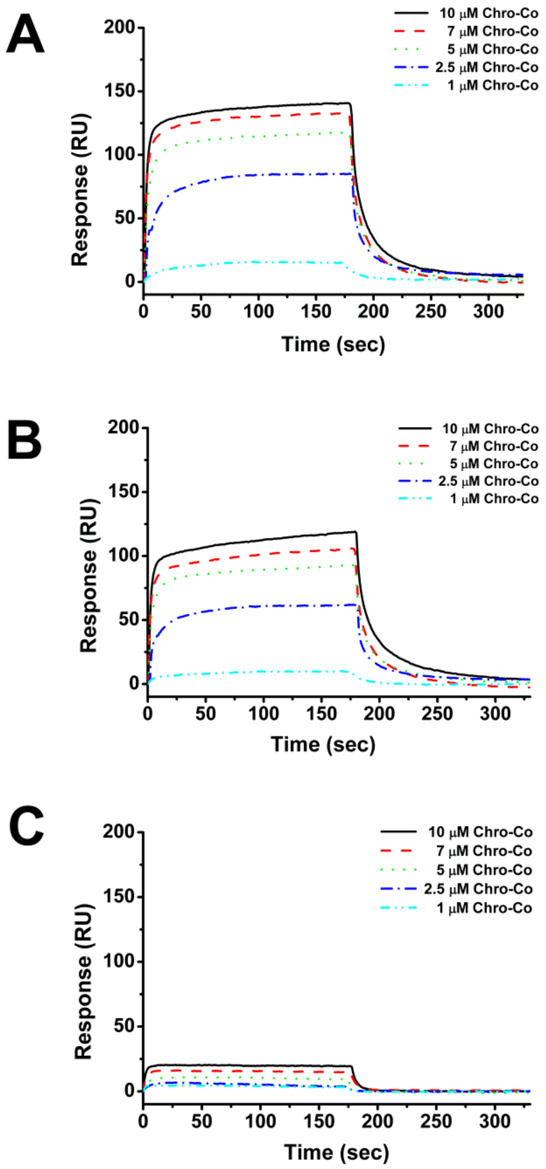

Figure S2. SPR sensorgrams show the interaction between immobilized 5'-biotin-labelled hairpin DNAs **(A)** CCG4, **(B)** CCG3, and **(C)** CCG2, and the various concentrations of target $Co^{II}(Chro)_2$ in 50 mM NaCl, buffered by 50 mM sodium cacodylate at pH 7.3. The resonance unit (RU) is defined as 1 RU = 1 pg/mm^2. Complexes obtained by subtracting the reference control are shown.

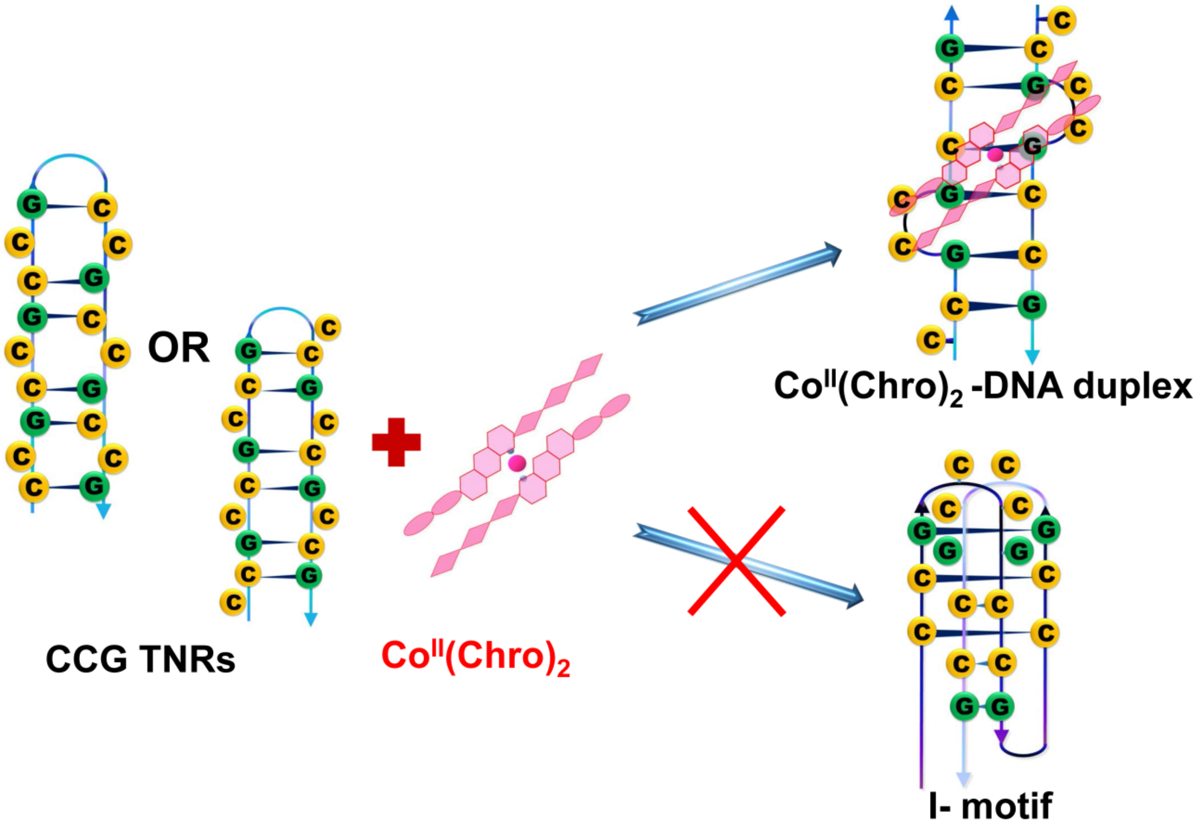

Figure S3. Schematic representation of $Co^{II}(Chro)_2$ complex showing the induction of the secondary structures to adopt the double helical conformation. In the absence of $Co^{II}(Chro)_2$, the secondary structures can form i-motifs.

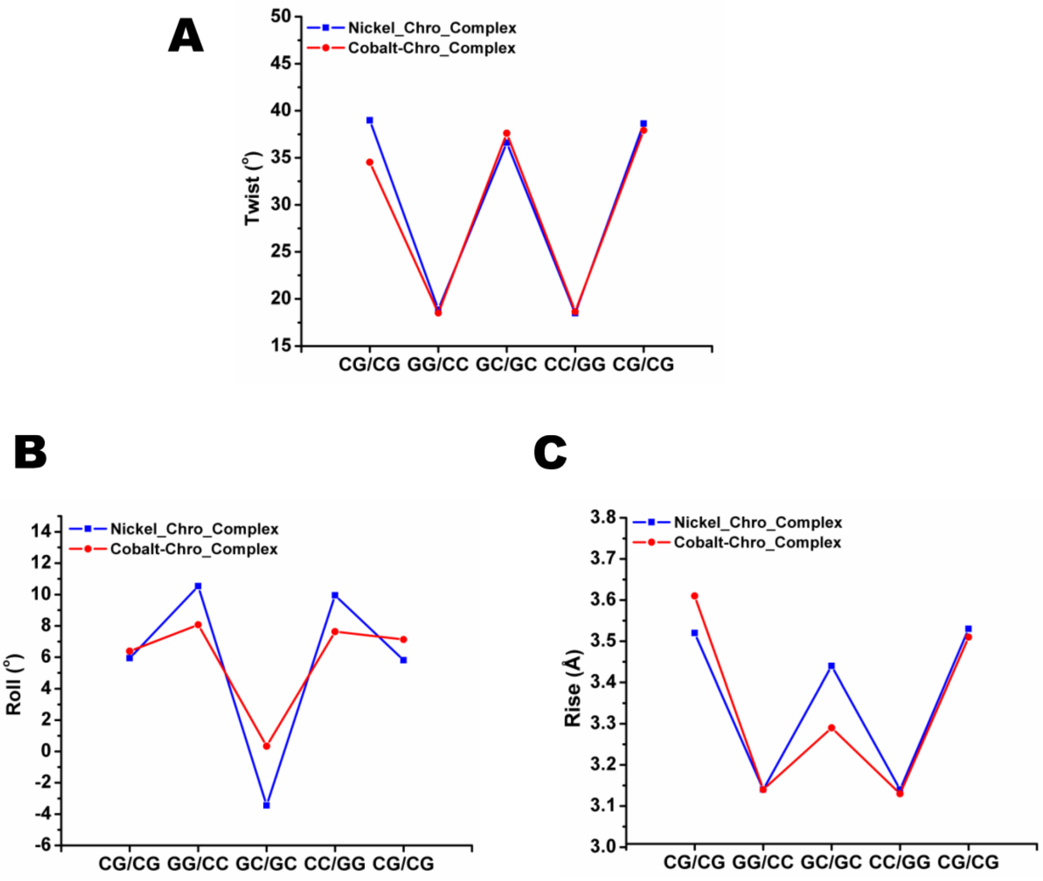

Figure S4. Comparison of DNA twist (A), roll (B) and rise (C) parameters of central GpGpCpC segment surrounding the Chro dimer binding site of the $Ni^{II}(Chro)_2$-$d(TT(CCG)_3AA)_2$ (PDB- 5XEW) and $Co^{II}(Chro)_2$-$d(TT(CCG)_3AA)_2$ (PDB- 5YZE).

Table S1. Crystallographic and refinement data of the dT(CCG)$_3$A and Co^{II}(Chro)$_2$-d[T(CCG)$_3$A]$_2$ complex structures.

Structure	dT(CCG)$_3$A Hairpin	Co^{II}(Chro)$_2$-d[T(CCG)$_3$A]$_2$ Complex		
**Crystallographic data**				
		**Inflection**	**Peak**	**High Remote**#
**Wavelength**	1.00000	1.60553	1.60482	1.56433
**Space group**	*P*4$_3$2$_1$2	*P*3$_2$12		
**a=b (Å)**	38.23	46.40	46.40	46.40
**c (Å)**	54.23	73.83	73.84	73.82
**α, β, γ (°)**	α=β=γ=90	α=β=90, γ=120	α=β=90, γ=120	α=β=90, γ=120
**Resolution (Å)***	30.00-1.71 (1.77-1.71)	30.00-1.92 (1.99-1.92)	30.00- 1.92 (1.99-1.92)	30.00-1.87 (1.94-1.87)
R_{merge}*	0.035 (0.474)	0.061 (0.192)	0.081 (0.240)	0.050 (0.171)
$I/\sigma I$*	33.92 (4.67)	57.894 (10.047)	77.804 (12.379)	59.890 (12.189)
**Completeness (%)***	99.6 (100.0)	99.6 (99.1)	99.6 (98.9)	99.5 (98.7)
**Multiplicity**	13.1 (12.9)	7.6 (7.1)	15.1 (13.3)	7.7 (7.3)
**Total reflections**	5789	54448	107623	59152
**Unique reflections**	4691	13571	13577	14686
**Refinement Data**				
**Resolution (Å)**	27.03-1.71	27.18- 1.87		
R_{factor}/R_{free}	0.227/0.285	0.1938/ 0.2518		
**r.m.s.d (Å)**	0.028	0.006		
**r.m.s.d (°)**	3.65	1.008		
**No. of Co^{2+} ions**	0	3		
**No. of waters**	60	77		
**PDB ID**	5DEV	5YZE		

**Outer shell statistics are shown in parentheses.*

#High Remote data is used for the structure refinement.

Table S2. Water-mediated interactions between DNA-DNA inter- or intra-strand in the two symmetrical dT(CCG)$_3$A hairpin structure.

DNA-DNA inter-strand				
**DNA strand 1**	**Distance (Å)**	**Water**	**Distance (Å)**	**DNA strand 2**
CYT2-O1P	2.6	W109	2.9	GUA7-OP1
CYT2-O5′	3.4	W109	2.9	CYT8-N4
CYT2-N4	3.0	W127	3.1	CYT9-O2P
CYT5-N3<br>CYT5-N4	3.5<br>3.0	W128	3.2	CYT5-O2P
GUA7-O2P	2.5	W106	3.3	GUA4-N2
CYT8-O2P	2.3	W102	3.5	CYT2-O2P
GUA10-N2	3.3	W133	3.4	ADE11-O4′
CYT3-O2<br>GUA4-O4′	3.5<br>3.4	W106	3.3	GUA4-N2
CYT9-N4<br>GUA10-N1	3.3	W133	3.4	ADE1-O4′

DNA-DNA intra-strand				
**DNA strand 1**	**Distance (Å)**	**Water**	**Distance (Å)**	**DNA strand 1**
GUA7-N2<br>CYT6-N4	3.0<br>3.6	W117	2.8	GUA7-N1
GUA7-O6′	2.7	W113	2.7	CYT6-O1P
GUA7-N7	2.7	W110	2.6	CYT6-O1P
GUA7-O′3	3.2	W132	3.5	CYT8-O1P
CYT8-O1P	2.8	W118	3.3	CYT9-O1P
THY1-O5′	2.9	W112	2.7	GUA4-O6
CYT3-O1P	3.0	W101	2.1	CYT8-O3′
CYT9-O3′	2.9	W123	3.2	CYT2-O4′
CYT9-N4	3.5	W133	3.3	GUA10-N2
THY1-O3′	3.4	W130	3.1	THY1-O4′
